# Gastrointestinal adenocarcinoma analysis identifies promoter methylation-based cancer subtypes and signatures

**DOI:** 10.1038/s41598-020-78228-y

**Published:** 2020-12-04

**Authors:** Renshen Xiang, Tao Fu

**Affiliations:** 1 Department of Gastrointestinal Surgery II, Renmin Hospital of Wuhan University, Wuhan, 430060 Hubei Province China; 2 The Central Laboratory of the First Clinical College of Wuhan University, Wuhan, 430060 Hubei Province China

**Keywords:** Cancer, Computational biology and bioinformatics

## Abstract

Gastric adenocarcinoma (GAC) and colon adenocarcinoma (CAC) are the most common gastrointestinal cancer subtypes, with a high incidence and mortality. Numerous studies have shown that its occurrence and progression are significantly related to abnormal DNA methylation, especially CpG island methylation. However, little is known about the application of DNA methylation in GAC and CAC. The methylation profiles were accessed from the Cancer Genome Atlas database to identify promoter methylation-based cancer subtypes and signatures for GAC and CAC. Six hypo-methylated clusters for GAC and six hyper-methylated clusters for CAC were separately generated with different OS profiles, tumor progression became worse as the methylation level decreased in GAC or increased in CAC, and hypomethylation in GAC and hypermethylation in CAC were negatively correlated with microsatellite instability. Additionally, the hypo- and hyper-methylated site-based signatures with high accuracy, high efficiency and strong independence can separately predict the OS of GAC and CAC patients. By integrating the methylation-based signatures with prognosis-related clinicopathologic characteristics, two clinicopathologic-epigenetic nomograms were cautiously established with strong predictive performance and high accuracy. Our research indicates that methylation mechanisms differ between GAC and CAC, and provides novel clinical biomarkers for the diagnosis and treatment of GAC and CAC.

## Introduction

According to epidemiological statistics, more than one in six deaths per year are caused by malignant tumors, a major disease currently threatening human health^1^. Gastrointestinal adenocarcinoma, the most common pathological type of gastrointestinal cancer, has contributed greatly to the threat^1^. The occurrence and progression of gastrointestinal adenocarcinoma are complex and slow processes involving multiple factors and steps. In addition to external factors such as diet, lifestyle and living environment, the pathogenesis of gastrointestinal adenocarcinoma also involves irreversible gene sequence changes and reversible epigenetic modifications^2^. In recent years, mutations in transcriptional profiles have been used to classify cancers into different subtypes and explore novel molecular markers that are related to different biological characteristics and survival outcomes^3–7^. Therefore, molecular-based pathogenic mechanisms and diagnostic markers of gastrointestinal adenocarcinoma subtypes have received extensive attention.

Epigenetics refers to heritable modifications manifested as changes in gene expression but not the DNA sequence, and these modifications play important roles in embryonic development, gene imprinting, cell differentiation and tumorigenesis^8^. The effects of epigenetic modifications in the tumor context include abnormal DNA methylation, histone modifications, activity of noncoding RNAs (such as microRNAs and lncRNAs), etc.^9^. Currently, abnormal DNA methylation, which is closely related to tumorigenesis and progression via the regulation of tumor suppressor genes, is the most thoroughly studied epigenetic modification^10^. More importantly, DNA methylation in gastrointestinal cancers has been extensively studied, especially CpG island methylation, which occurs in 56% of the protein-coding genes in the human genome^10,11^, and the diagnostic potential of certain CpG methylation sites for gastrointestinal cancer has been effectively assessed^12,13^. Moreover, several markers for overall survival (OS) of patients with gastrointestinal cancer have been developed according to transcription and methylation profiles, but these studies were based mostly on analysis of candidate genes and focused mainly on CpG island methylation and its relationship with gene expression^14–18^. However, the DNA methylation-based cancer subtypes and prognostic signatures for gastrointestinal adenocarcinoma have not been fully investigated.

**Figure 1:**
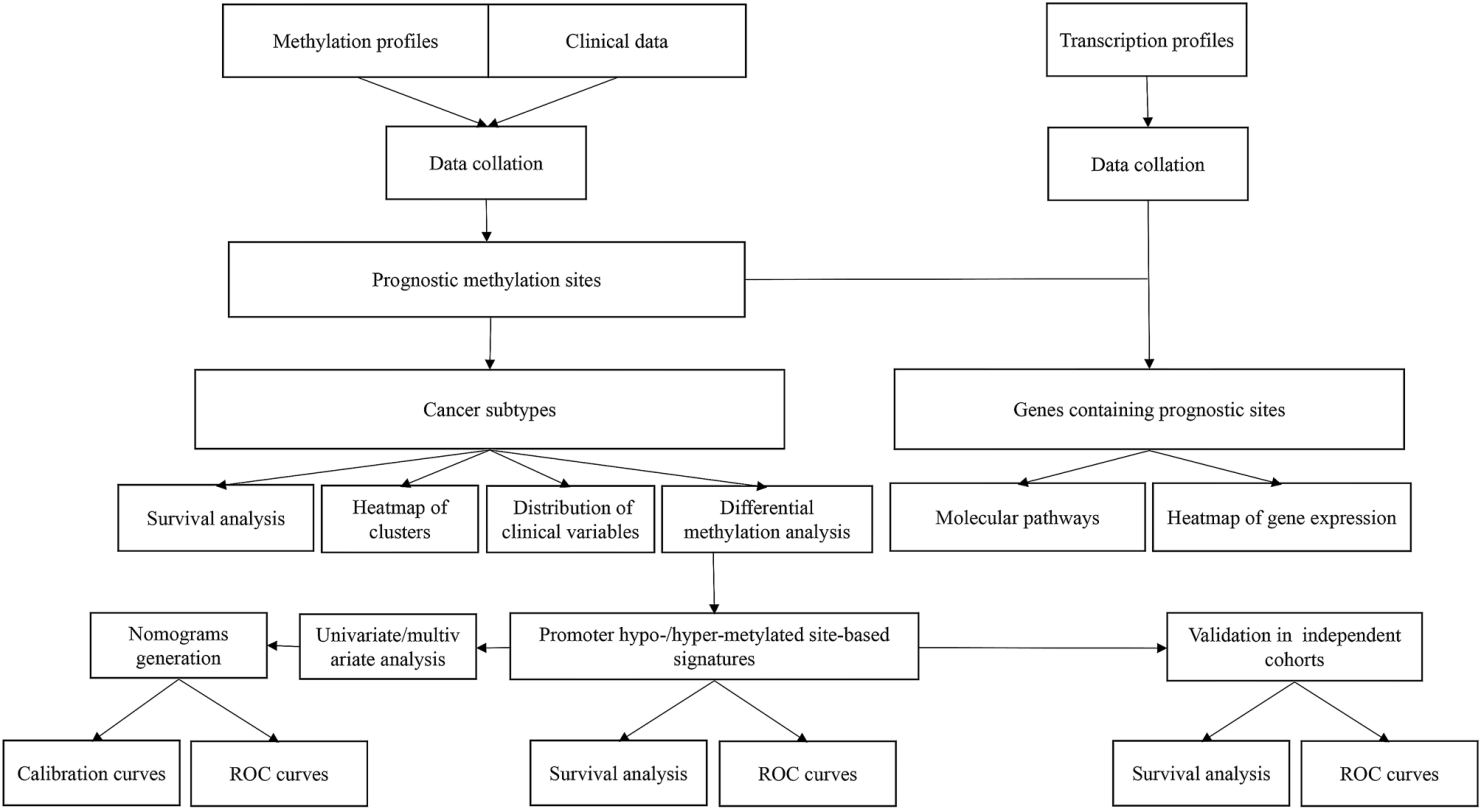
The detailed process of the present study.

In this study, the promoter methylation profiles were accessed from open public databases to classify gastric adenocarcinoma (GAC) and colon adenocarcinoma (CAC) subtypes. The prognostic risk scoring signatures based on promoter hypo- or hyper-methylated sites were constructed for GAC and CAC. Additionally, we explored the relationship between gene expression and methylation levels, and investigated the signaling pathways involving genes containing independent prognostic methylation sites (Fig. 1).

## Results

### Promoter methylation patterns reveal cancer subtypes

**Figure 2:**
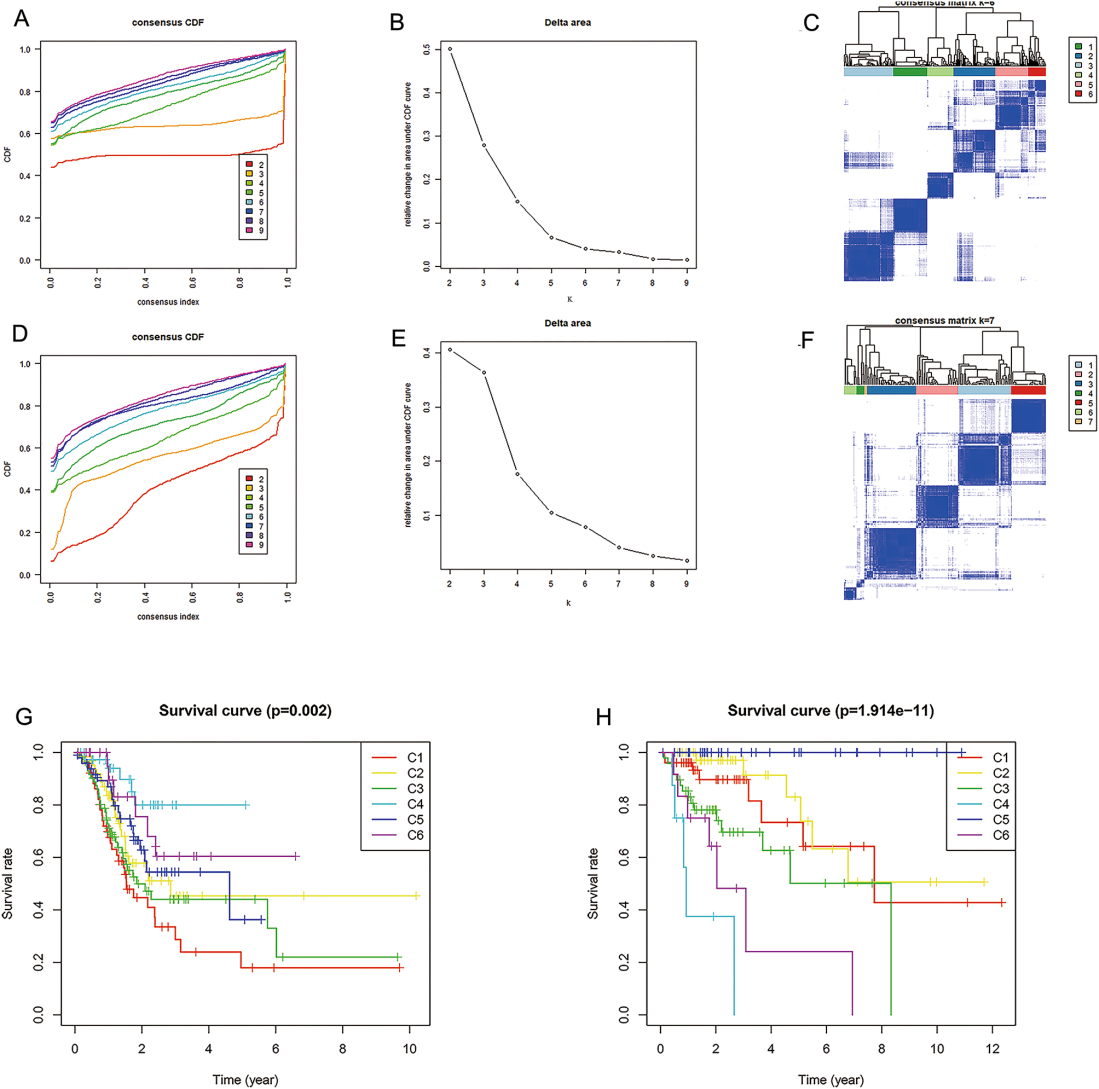
Promoter methylation-based cancer subtypes. (**A–C**) GAC was divided into 6 clusters at a clustering threshold of maxK = 6. (**D–F**) 6 major clusters were identified from CAC when maxK = 7. (**G**) Kaplan–Meier analysis indicated that clusters 4 and 1 separately showed the best and worst OS profiles in GAC. (**H**) Kaplan–Meier analysis indicated that clusters 5 and 4 revealed the best and worst OS profiles in CAC, respectively. *GAC* gastric adenocarcinoma, *CAC* colon adenocarcinoma.

Multivariate analysis revealed a total of 131 independent prognostic methylation sites [68 for GAC (Supplementary Table 1) and 63 for CAC (Supplementary Table 2)], which were used to identify GAC and CAC subtypes. According to promoter methylation-based consensus clustering, six clusters that contained all the GAC samples were identified at a clustering threshold of maxK = 6 (Fig. 2A–C). In addition, six major CAC clusters that contained 98.98% of the CAC samples were identified for maxK = 7 (Fig. 2D–F), cluster 7 was excluded because it contains only 2 samples. The Kaplan–Meier survival curves (Fig. 2G,H) determined the statistical significance of the consensus clustering results for GAC and CAC (*P* = 0.002 and 1.914e−11, respectively). In GAC, clusters 4 and 1 showed the best and worst OS profiles, while in CAC, clusters 5 and 4 revealed the best and worst OS profiles, respectively.

### Differential methylation sites across clusters

**Figure 3:**
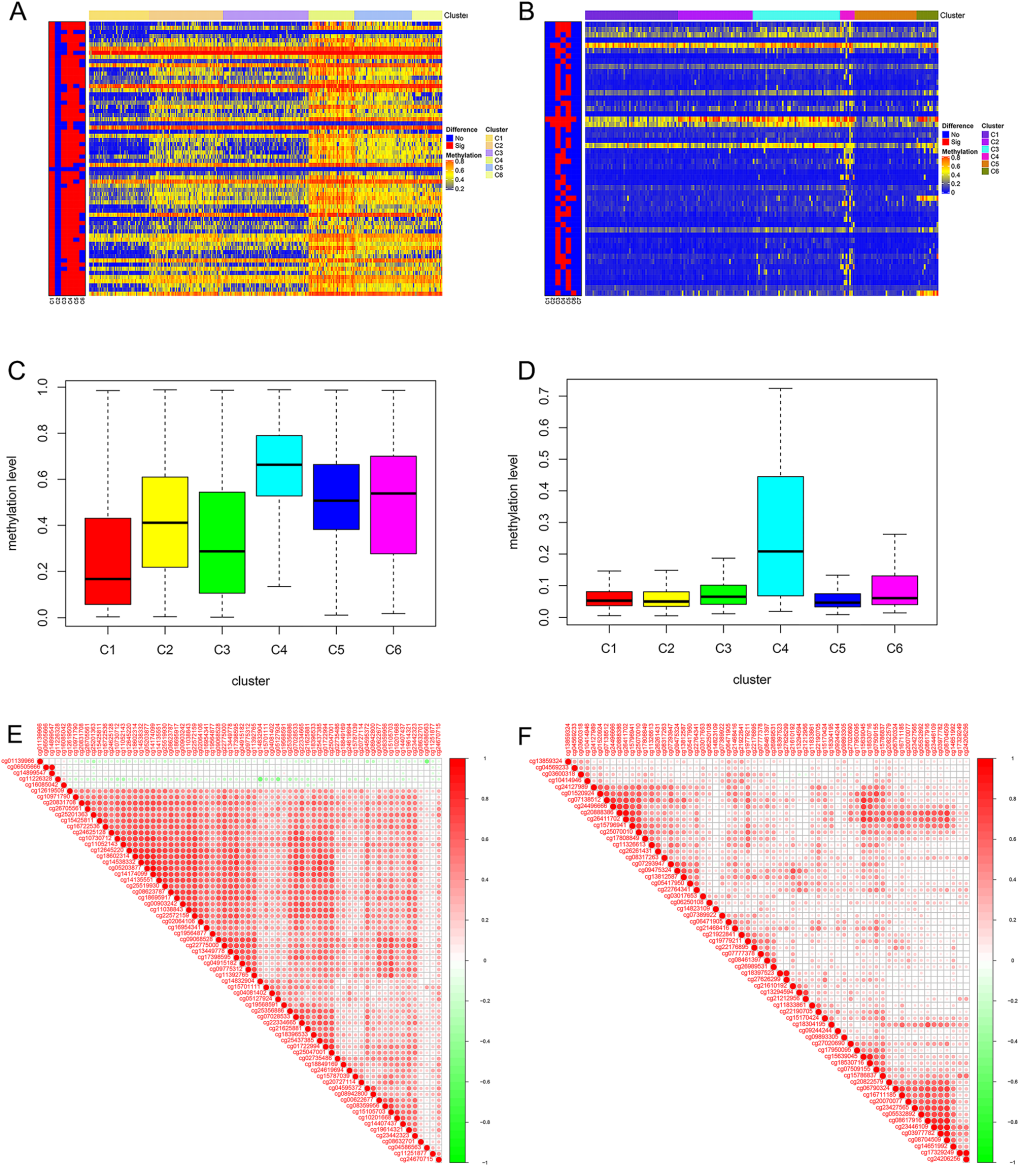
Differential analysis of independent prognostic methylation sites across clusters, and the co-methylation pattern of independent prognostic methylation sites. (**A**,**B**) showed the heatmaps of differential methylation sites in GAC and CAC, respectively. (**C**) Cluster 1 was hypo-methylated across six clusters in GAC. (**D**) Cluster 4 was hyper-methylated in CAC, while no obvious difference was found between the remaining clusters. (**E**) In GAC, 5 methylation sites were negatively correlated with the other 63 methylation sites, while the other 63 methylation sites showed positive correlation or no co-methylation relationship. (**F**) In CAC, 63 methylation sites showed significant positive correlation or no methylation relationship. *GAC* gastric adenocarcinoma, *CAC* colon adenocarcinoma.

Considering the remarkable impact of the classification scheme on patients’ prognosis, the differential methylation sites among clusters were investigated. Difference analysis confirmed the significant differences of promoter methylation across clusters for both GAC and CAC (Fig. 3A,B). In GAC, cluster 1 was hypo-methylated, while cluster 4 was more methylated than others (Fig. 3C). Considering the survival analysis (Fig. 2G), the OS of patients became worse with the decrease of promoter methylation level. Therefore, promoter hypomethylation is unfavorable to the OS of GAC patients. In CAC, cluster 4 was hyper-methylated, while no significant difference was found among other clusters (Fig. 3D). More importantly, cluster 4 showed the worst OS compared with other clusters (Fig. 2H), which indicated that promoter hypermethylation is detrimental to the OS of CAC patients.

### Co-methylation pattern and methylation levels of independent prognostic sites

In this study, the co-methylation patterns of 68 independent prognostic methylation sites in GAC and 63 methylation sites in CAC were discussed by correlation analysis. In GAC, 5 methylation sites were negatively correlated with the other 63 methylation sites, while the other 63 methylation sites were positively correlated or had no co-methylation relationship (Fig. 3E). In CAC, 63 methylation sites showed significant positive correlation or no methylation relationship (Fig. 3F). Additionally, according to the differential methylation analysis, 65 differentially methylated sites were obtained in GAC, including 63 hypo-methylated sites and 2 hyper-methylated sites (Supplementary Table 3). 19 differential methylation sites were identified in CAC, consisting of 18 hyper-methylated sites and 1 hypo-methylated site (Supplementary table 4). Therefore, this study mainly captured hypomethylation sites in GAC, while hypermethylation sites in CAC.

### Microsatellite instability levels across clusters

In GAC, cluster 4 has the highest microsatellite instability (MSI), followed by cluster 5, while the other clusters showed a low MSI, including the hypo-methylated cluster 1 (Supplementary Fig. 1A). In CAC, the mean of MSI in the hyper-methylated cluster 4 was lower than the other five clusters, even if the difference in MSI between six clusters was not obvious (Supplementary Fig. 1B). These results indicated that hypomethylation in GAC and hypermethylation in CAC are negatively correlated with MSI.

### Proportion of clinicopathologic variables in cancer subtypes

**Figure 4:**
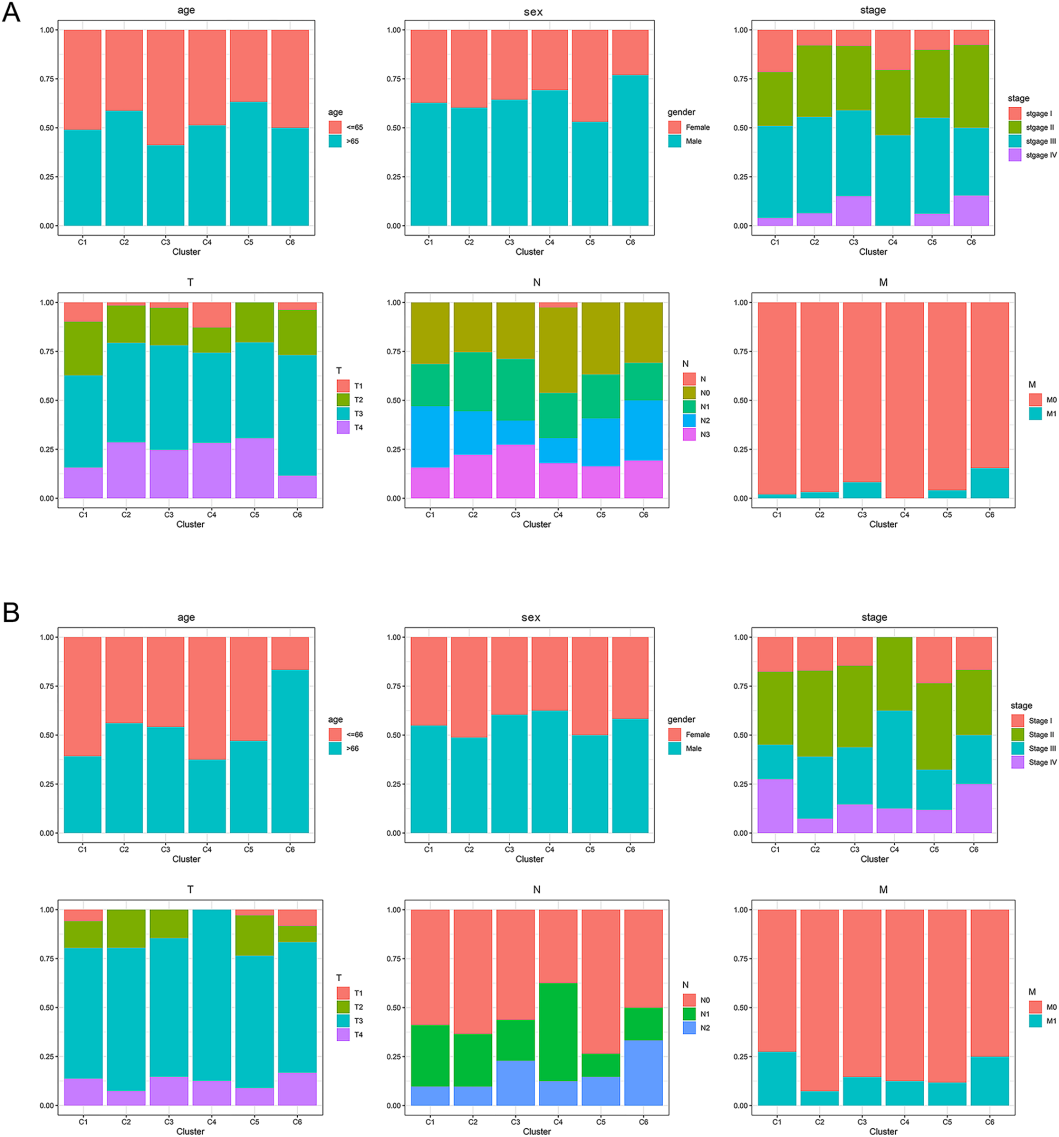
Proportion of clinicopathologic features in cancer subtypes. (**A**) In GAC, there was no significant difference in the proportion of age, sex, T stage and N stage across six cluster. M1 and stage IV were absent in cluster 4, while increased in other hypo-methylated clusters. (**B**) In CAC, there was no significant difference in the proportion of sex and M stage in six clusters; the patients were older in cluster 6; in cluster 4, stage I and T1/2 were absent, and N1 was less, while stage II/III/IV, T3/4 and N1/2 accounted for more. GAC: gastric adenocarcinoma, CAC: colon adenocarcinoma.

The proportion of clinicopathological variables (including age, sex, grade, and pathological stage) was investigated in each cluster. In GAC, there was no significant difference in the proportion of age, sex, T stage and N stage across six cluster. M1 and stage IV were absent in cluster 4, while increased in other hypo-methylated clusters, which indicated that promoter hypomethylation can accelerate GAC progression (Fig. 4A). In CAC, there was no significant difference in the proportion of sex and M stage in six clusters; the patients were older in cluster 6; in cluster 4, stage I and T1/2 were absent, and N1 was less, while stage II/III/IV, T3/4 and N1/2 accounted for more (Fig. 4B), which proved that promoter hypermethylation can promote the malignant progression of GAC.

### Gene expression and molecular pathways

**Figure 5:**
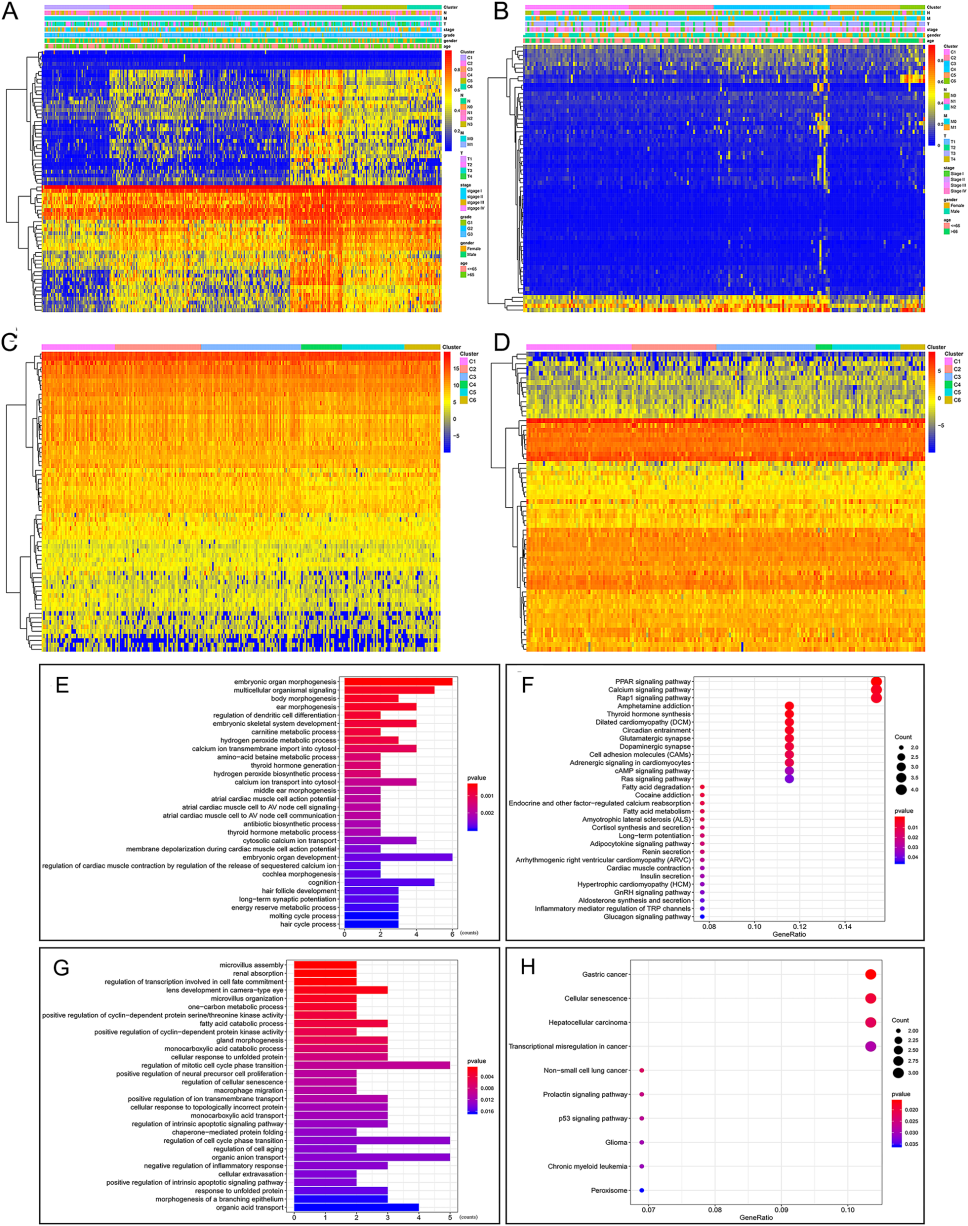
The relationship between promoter methylation and gene expression, and molecular pathway enrichment analysis. (**A**) The methylation profiles of 68 independent prognostic methylation sites in GAC. (**B**) The methylation profiles of 63 independent prognostic methylation sites in CAC. (**C**) The expression levels of genes containing 68 independent prognostic methylation sites in GAC. (**D**) The expression levels of genes containing 63 independent prognostic methylation sites in CAC. (**E**) The top 30 BPs in GAC. (**F**) The top 30 KEGG pathways in GAC. (**G**) The top 30 BPs in CAC. (**H**) The top 10 KEGG pathways in CAC. *GAC* gastric adenocarcinoma, *CAC* colon adenocarcinoma, *BP* biological processe, *KEGG* Kyoto Encyclopedia of Genes and Genomes.

The methylation profiles of the GAC and CAC clusters were shown in Fig. 5A,B, respectively. To elucidate the effect of methylation on gene expression, the genes with independent prognostic methylation sites were explored, and their expression levels were visualized via unsupervised hierarchical clustering (Fig. 5C,D), which showed negative correlation between gene expression and promoter methylation. Additionally, molecular functional analysis showed that hypomethylation in GAC was associated with substance metabolism (e.g. hydrogen peroxide metabolic process, cytosolic calcium ion transport and amino-acid betaine metabolic process), ferroptosis (e.g. fatty acid oxidation, lipid oxidation and fatty acid degradation) and well-known cancer-related pathways (e.g. Ras signaling pathway, Rap1 signaling pathway and calcium signaling pathway) (Fig. 5E,F). Hypermethylation in CAC involved various types of pathways, including carcinogenic pathway (e.g. p53 signaling pathway), cell cycle (e.g. regulation of mitotic cell cycle phase transition and regulation of cell cycle phase transition), ferroptosis (e.g. peroxisome and fatty acid catabolic process), anion transport (e.g. positive regulation of ion transmembrane transport), cell senescence (e.g. cellular senescence, regulation of cellular senescence and regulation of cell aging), catabolism (e.g. fatty acid catabolic process and monocarboxylic acid catabolic process), inflammation (e.g. negative regulation of inflammatory response) and apoptosis (e.g. regulation of intrinsic apoptotic signaling pathway), etc., (Fig. 5G,H).

### Generation and validation of hypo-methylated site-based signature for GAC

**Figure 6:**
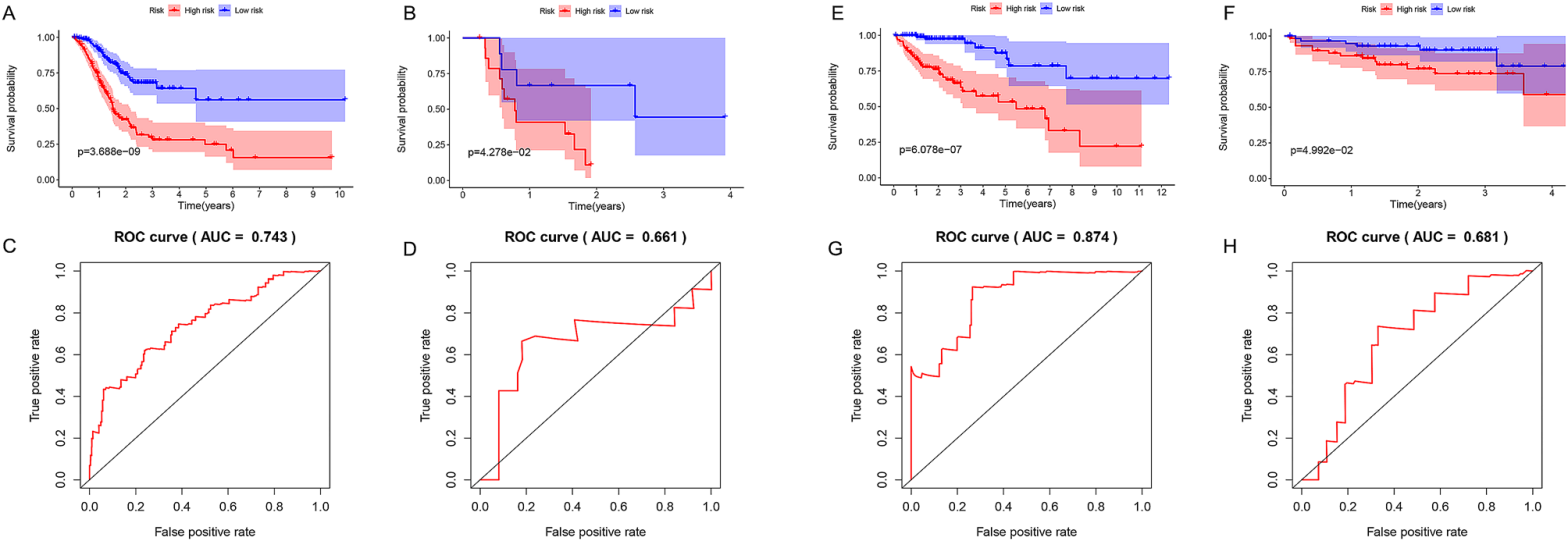
Generation, evaluation and validation of hypo-/hyper-methylated site-based signature. (**A,B**) In GAC, the OS of the high-risk group in training and validation cohorts is significantly worst (training cohort: *P* = 3.688e−09, validation cohort: *P* = 4.278e−02). (**C,D**) In GAC, the AUCs of ROC curves in training and validation cohorts for predicting OS are 0.743 and 0.661, respectively. (**E,F**) In CAC, the OS of the high-risk group in training and validation cohorts is significantly worst (training cohort: *P* = 6.078e−07, validation cohort: *P* = 4.992e−02). (**G,H**) In CAC, the AUCs of ROC curves in training and validation cohorts for predicting OS are 0.874 and 0.681, respectively. *GAC* gastric adenocarcinoma, *CAC* colon adenocarcinoma, *OS* overall survival, *AUC* area under the curve, *ROC* receiver operating characteristic.

According to the difference analysis, since GAC is dominated by hypomethylation, 63 hypo-methylated sites were enrolled into Lasso Cox regression analysis to construct a prognostic risk scoring signature for GAC. And a 16-hypomethylation site-based signature for OS was identified, and the RS for each patient could be calculated on the basis of the methylation levels of 16 hypo-methylated sites and the relative coefficients (Table 1): RS = (2.52 * cg01139966) + (2.05 * cg04595372) + (− 2.21 * cg08632701) + (− 2.19 * cg08942800) + (2.37 * cg11052143) + (5.78 * cg11226328) + (− 2.45 * cg11251877) + (− 1.16 * cg14174099) + (− 1.77 * cg14832904) + (− 1.73 * cg17398595) + (− 1.167 * cg18849169) + (− 1.17 * cg19568591) + (− 1.58 * cg20727114) + (1.74 * cg20831708) + (1.20 * cg25519930) + (− 1.60 * cg26705561). Considering 10 coefficients are less than 0, which indicated that 10 hypo-methylated sites are protective markers and the remaining 6 are hazardous factors. In the training cohort (Illumina Human Methylation 450 platform) and validation cohort (Illumina Human Methylation 27 platform), Kaplan–Meier analysis for GAC (Fig. 5A,B) demonstrated more favorable OS in the low-risk group than in the high-risk group (training cohort: *P* = 3.688e−09, validation cohort: *P* = 4.278e−2). According to the ROC curves, the area under the curve (AUC) for the 16-hypomethylation site-based signature was 0.743 in the training cohort (Fig. 6C), while AUC was 0.661 in the validation cohort (Fig. 6D), revealing the high accuracy and efficiency of the prognostic signature.

**Table 1:** Information on 16 methylation sites used to construct the prognostic model.

Sites	Relative coefficient	HR	*P* value^a^
cg01139966	2.523686109	12.47	4.05E−05
cg04595372	2.046696618	7.74	2.20E−04
cg08632701	− 2.214986058	0.11	4.74E−04
cg08942800	− 2.185192503	0.11	5.35E−05
cg11052143	2.37032886	10.70	7.31E−05
cg11226328	5.784881598	325.34	8.09E−04
cg11251877	− 2.452567914	0.09	2.74E−05
cg14174099	− 1.158703109	0.31	1.52E−04
cg14832904	− 1.767649559	0.17	9.23E−05
cg17398595	− 1.732461009	0.18	6.04E−04
cg18849169	− 1.166925185	0.31	1.86E−05
cg19568591	− 1.174057641	0.31	6.24E−04
cg20727114	− 1.581217147	0.21	9.53E−05
cg20831708	1.738078564	5.69	1.17E−04
cg25519930	1.198822182	3.32	6.05E−04
cg26705561	− 1.604620464	0.20	1.98E−05

^a^Derived from multivariate Cox regression analysis.

### Generation and validation of hyper-methylated site-based signature for CAC

The differential methylation analysis showed that CAC was dominated by hypermethylation, 18 hyper-methylated sites were included in the Lasso Cox regression to generate a hyper-methylated site-based prognostic risk scoring signature. And a 12-hypermethylated site-based signature was generated for predicting the OS of CAC patients (Table 2). RS = (3.02 * cg03017653) + (22.84 * cg03977782) + (3.00 * cg05417950) + (36.54 * cg06250108) + (2.18 * cg09893305) + (3.12 * cg10414946) + (3.06 * cg15170424) + (5.77 * cg15639045) + (-9.51 * cg15786837) + (22.02 * cg17329249) + (8.12 * cg21212956) + (− 23.31 * cg24206256) (Table 2). Considering 10 coefficients are greater than 0, which indicated that 10 hyper-methylated sites are hazardous markers and the remaining 2 are protective factors. The Kaplan–Meier survival curves (Fig. 6E–F) revealed more favorable OS in the low-risk group than in the high-risk group (training cohort: *P* = 6.078e−7, validation cohort: *P* = 4.992e−2). Besides, the AUC of ROC curves was 0.874 in the training cohort (Fig. 6G), while AUC was 0.681 in the validation cohort (Fig. 6H), indicating the high accuracy and efficiency of the hyper-methylated site-based signature.

**Table 2:** Information on 12 methylation sites used to construct the prognostic model.

Sites	Relative coefficient	HR	*P* value^a^
cg03017653	3.016921603	20.43	9.64E−05
cg03977782	22.84228682	8.32E+09	3.51E−05
cg05417950	2.999350491	20.07	1.27E−04
cg06250108	36.53775238	7.38	8.91E−04
cg09893305	2.179272838	8.84	2.52E−04
cg10414946	3.115359352	22.54	5.98E−04
cg15170424	3.06373655	21.41	1.38E−06
cg15639045	5.766452521	319.40	1.39E−04
cg15786837	− 9.508755839	7.42E−05	1.30E−07
cg17329249	22.02401981	3.67E+09	1.64E−04
cg21212956	8.120594101	3.36E+03	2.34E−04
cg24206256	− 23.31386822	7.50E−11	2.45E−04

^a^Derived from multivariate Cox regression analysis.

### Construction and evaluation of nomogram for GAC

**Figure 7:**
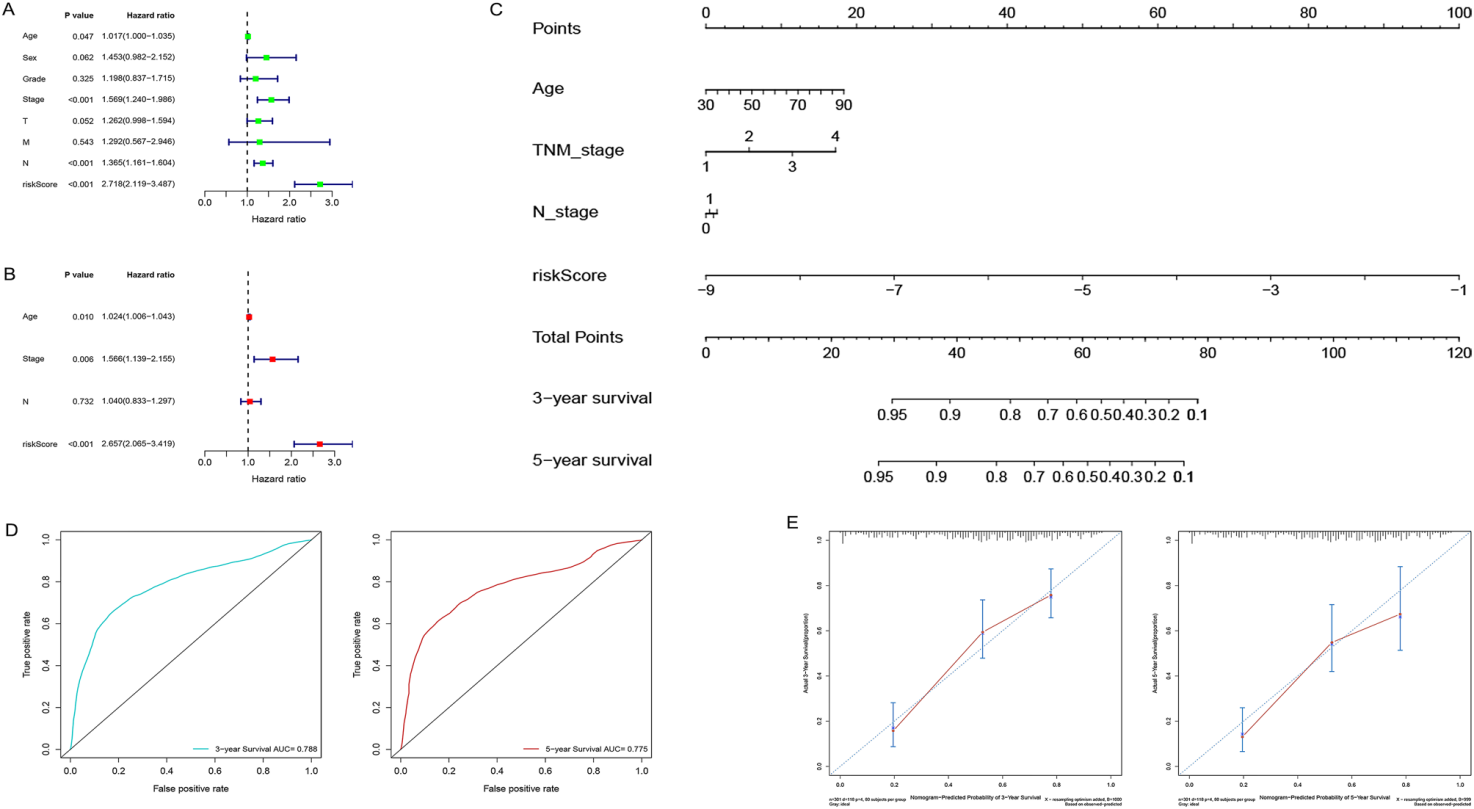
Construction and evaluation of nomogram for GAC. (**A**) Univariate analysis revealed that the patient’s age, TNM stage, N stage and risk score jointly affect the prognosis of GAC patients (all *P* < 0.05). (**B**) Multivariate analysis indicated that age, TNM stage and risk score are independent prognostic factors for GAC patients (all *P* < 0.05). (**C**) A nomogram combined prognosis-related clinicopathologic variables and risk score for predicting 3-year and 5-year OS of GAC patients. (**D**) The AUCs of ROC curves for predicting 3-year and 5-year OS are 0.788 and 0.775, respectively. (**E**) The calibration curves for predicting 3-year and 5-year OS are in good agreement with the actual observations. *GAC* gastric adenocarcinoma, *AUC* area under the curve, *ROC* receiver operating characteristic, *OS* overall survival.

The study carried out a univariate analysis of the clinicopathologic characteristics and RS in the training cohort, which revealed that the patient’s age, TNM stage, N stage and RS jointly affect the prognosis of GAC patients (Fig. 7A, all *P* < 0.05). The older the age, the later the stage; and the higher the RS, the worse the prognosis of patients. Additionally, multivariate analysis indicated that age, TNM stage and RS are independent prognostic factors for GAC patients (Fig. 7B, all *P* < 0.05). Subsequently, four prognosis-related factors were combined, and a nomogram was constructed to predict OS (Fig. 7C). The AUCs of ROC curves for predicting 3-year and 5-year OS are 0.788 and 0.775, respectively (Fig. 7D), and the calibration curves for predicting 3-year and 5-year OS are in good agreement with the actual observations (Fig. 7E).

### Construction and evaluation of nomogram for CAC

**Figure 8:**
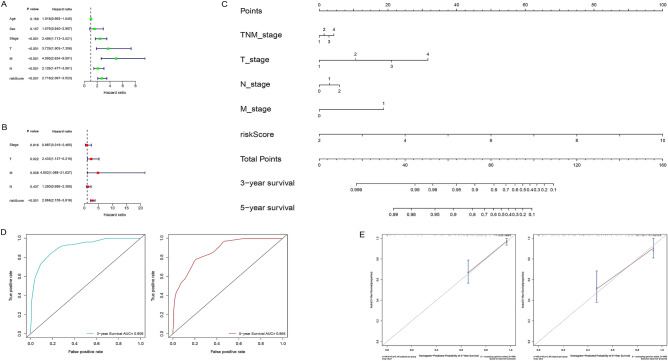
Construction and evaluation of nomogram for CAC. (**A**) Univariate analysis demonstrated that patients’ TNM stage, T stage, N stage, M stage and risk score jointly impact on the prognosis of CAC patients (all *P* < 0.05). (**B**) Multivariate analysis showed that T stage, M stage and RS retained independent predictive ability (all *P* < 0.05). (**C**) A nomogram containing five prognosis-related factors for predicting 3-year and 5-year OS of CAC patients. (**D**) The AUCs of ROC curves for predicting 3-year and 5-year OS are 0.908 and 0.864, respectively. (**E**) The calibration curves for predicting 3-year and 5-year OS are in good agreement with the actual observations. *CAC* colon adenocarcinoma, *AUC* area under the curve, *ROC* receiver operating characteristic, *OS* overall survival.

In univariate analysis, TNM stage, T stage, N stage, M stage and RS have impacts on the prognosis of CAC patients (Fig. 8A, all *P* < 0.05). The later the clinicopathologic stage and the higher the RS, the worse the patient’s OS. Multivariate analysis showed that T stage, M stage and RS retained independent predictive ability (Fig. 8B, all *P* < 0.05). Afterwards, five prognosis-related factors were enrolled into construction of a nomogram for predicting 3-year and 5-year OS of CAC patients (Fig. 8C). The AUCs of ROC curves for predicting 3-year and 5-year OS are 0.908 and 0.864, respectively (Fig. 8D), and calibration curves showed that the nomogram prediction effect is excellent (Fig. 8E).

## Discussion

Gastrointestinal adenocarcinoma is the most common pathological type of gastrointestinal cancer, with high incidence and mortality; this disease seriously endangers human health, and its pathogenesis involves a complex process of accumulation of classical DNA sequence changes and epigenetic modifications^10^. DNA methylation can affect gene transcription and expression via various mechanisms, such as interfering with transcription factors, recruiting histones, and altering chromatin structure. Abnormal DNA methylation manifests mainly as a decrease in the genome-wide methylation level and promoter hypermethylation; the former can lead to proto-oncogene activation, loss of imprinting and chromosome instability, while the latter can silence the expression of tumor suppressor genes, cell cycle regulatory genes and apoptotic genes^11,19,20^. Promoter DNA methylation refers to the selective addition of a methyl group to cytosines in CpG sequences to form 5-methylcytosine via a reaction catalyzed by methyltransferases^21^. This event plays a vital role in epigenetic modification and can regulate gene expression without changing the DNA sequence^21^. CpG sequences are distributed unevenly across the human genome and are often clustered in CpG islands, which are located mostly in the promoter and first exon regions and are found in 56% of human protein-coding genes^10^. Currently, numerous genes with promoter methylation have been identified in gastrointestinal cancer and are related to tumorigenesis, progression and prognosis^22^. Therefore, the therapeutic targets and prognostic biomarkers for gastrointestinal adenocarcinoma at the epigenetic level (e.g., DNA methylation level) remain to be investigated. Several studies have identified gene-specific DNA methylation signatures for OS in gastrointestinal cancers^14–17^, but no study has classified cancer subtypes or constructed prognostic signatures for gastrointestinal adenocarcinoma based on promoter DNA methylation sites. Here, we assessed the DNA methylation profile and corresponding clinical information of patients with gastrointestinal adenocarcinoma from publicly available databases and identified promoter methylation-based cancer subtypes, as well as hypo- and hyper-methylated site-based signatures for predicting OS of GAC and CAC patients.

In recent years, with the rapid development of gene arrays and sequencing technique, the idea of molecular typing has emerged quietly. Molecule-based cancer subtypes carry unique genomic characteristics, which provides accurate diagnosis and treatment of gastrointestinal cancers. Previously well-known molecular subtyping in GAC is TCGA subtypes^23^ and Asian Cancer Research Group (ACRG) subtypes^24^, in which TCGA typing is composed of Epstein Barr Vims positive, MSI, genomically stable (GS) and chromosomal instability (CIN), while ACRG contains MSI, MSS/EMT, MSS/TP53 + and MSS/TP53-. The detailed clinicopathological and molecular characteristics of each subtype within the two molecular typing are shown in reference^25^ Both molecular typing identified MSI characterized by high-frequency mutations and the best prognosis, but the other 3 subtypes in the two molecular typing were partially overlaping. For instence, GS and CIN of TCGA exist in all ACRG subtypes; GS of TCGA is not equal to MSS/EMT of ACRG based on the mutation frequency of CDH1 and RHOA; TCGA typing does not involve hypo-methylated sites, and the samples of ACRG typing are all Asian populations. Therefore, the existing molecular typing in GAC is not perfect, and the idea of individualized treatment of GAC based on molecular subtyping is just emerging and worthy of further exploration. In this study, six hypo-methylated clusters for GAC were generated with different OS profiles, the patients’ OS and tumor progression become worse as the methylation level decreases, and MSI was inversely proportional to hypomethylation. Hypomethylation can promote the up-regulation of proto-oncogenes or tumor progression-related genes through various pathways, thereby changing the biological behavior of cancer^26–28^, which is consistent with our results. Currently, people are generally keen to study the phenomenon of hypermethylation in cancer. However, the effect of hypomethylation on tumor biological behavior and prognosis is rarely explored. Therefore, our results lay a strong foundation for the research of hypomethylation in GAC.

In 2012, CAC was divided into three subtypes (namely TCGA typing: CIN, high mutation and ultra-high mutation) based on whole exome sequencing^29^. Afterwards, a variety of molecular typing was proposed based on gene mutation, copy number, non-coding RNA, and proteomics, etc.^30–35^. Different molecular typing was related to the clinicopathologic characteristics and prognosis of patients, but the pattern was not observed. Until 2015, the Cancer Subtyping Consortium (CRCSC) put forward a new classification method (consensus molecular subtype, CMS), classifying CAC into CMS1, CMS2, CMS3 and CMS4, with different clinicopathologic and molecular characteristics^36^. CMS typing is the most recognized molecular typing in the world, integrating the most data. In view of the multiple factors affecting tumorigenesis and malignant progression, further research is needed to improve the existing molecular typing. In this study, six hyper-methylated clusters were generated for CAC, with distinct OS profiles, clinicopathologic features and MSI level, revealing that promoter hypermethylation is closely associated with the malignant progression of CAC, which is consistent with the previous studies^37^. Therefore, our results can be used as an important supplement to CAC molecular typing.

To explore the molecular mechanism of independent prognostic methylation sites involved in tumorigenesis, genes containing methylation sites were extracted. Their expression was negatively correlated with their promoter methylation, consistent with the general view that promoter hypomethylation usually causes gene up-regulated, while hypermethylation silences gene expression^38–40^. Additionally, molecular functional analysis revealed that hypomethylation in GAC was closely related to substance metabolism, ferroptosis, Ras signaling pathway, while hypermethylation in CAC involved in p53 signaling pathway, cell cycle, ferroptosis, anion transport, cell senescence, catabolism, inflammation and apoptosis, etc. These results indicated that genes in the these pathways may be potential therapeutic or prognostic targets for GAC and CAC by regulating their activity. A scan of the published literature revealed that some results support our observations. For instance, approximately 40%-50% of sporadic colorectal cancer cases exhibit P53 mutations^7,41^, which play a decisive role in tumor biological behavior. The mutation of P53 is related to lymphatic invasion of proximal colon cancer, and to lymphatic and vascular invasion of distal colon cancer^42^. Additionally, the mutant showed stronger drug resistance and poorer prognosis than the wild type^43^. Although the roles of these enrichment pathways or genes in the pathways in GAC and CAC have not been fully confirmed, there is evidence that they are associated with GAC and CAC carcinogenesis.

Since the promoter methylation-based cancer subtypes show distinct OS profiles, suggesting that the classification based on promoter methylated sites can be used to predict patients’ OS. Therefore, the hypo- and hyper-methylated site-based signatures with high accuracy, high efficiency and strong independence were established, which can separately predict the OS of GAC and CAC patients. Two promoter methylation-based predictive signatures involving 791 samples were identified from two cohorts and validated though two independent cohorts though Lasso Cox regression, which can identify the combination of promoter methylation sites with the best predictive power. Moreover, two nomograms combining RS and prognosis-related clinicopathologic variables provide a visual method to predict the OS of patients, which is more accurate and effective than using signature alone, which can guide individualized treatment of clinical decision-making.

However, the study had limitations. Firstly, robust clinical and experimental research is necessary to gain more insight into the modulatory roles of promoter methylation on gene activity and the crucial effects of regulated genes in the corresponding pathways. Secondly, despite the high accuracy and predictive performance of the nomogram, other prognostic clinical parameters of the patients can not be obtained from databases, so the variables involved in the nomogram are limited, and further improvement is needed in the later stage. Thirdly, the sample size of rectal adenocarcinoma in the TCGA database is limited, so this research has not been downloaded and analyzed yet.

## Conclusion

The OS profiles and tumor progression became worse as the methylation level decreased in GAC or increased in CAC, and hypomethylation in GAC and hypermethylation in CAC were negatively correlated with MSI. The hypo- and hyper-methylated site-based signatures with high accuracy, high efficiency and strong independence can separately predict the OS of GAC and CAC patients, and two nomograms combined RS and prognosis-related clinicopathologic characteristics provide an intuitive and accurate method for predicting patients’ OS. Our research indicated that methylation mechanisms differ between GAC and CAC, and provided novel clinical biomarkers for the diagnosis and treatment of GAC and CAC. Considering the limitations of our study, future experimental studies will facilitate the extension of these findings.

## Materials and methods

### Acquisition and processing of publicly available data from open public databases

The detailed process of this study is shown in Fig. 1. The DNA methylation profiles (Illumina Human Methylation 450 platform) of gastrointestinal adenocarcinoma patients were accessed from the UCSC Xena platform (http://xena.ucsc.edu/). The corresponding clinical information (including age, sex, tumor grade, tumor-node-metastasis [TNM] stage, survival time and survival status) of samples were downloaded from publicly available the Cancer Genome Atlas (TCGA) database (http://cancergenome.nih.gov/). A total of 791 tumor (406 GAC and 385 CAC) samples, which detailed clinicopathologic features were listed in Table 3, were enrolled in the study after processing of the original data with Perl software. The abovementioned samples were matched with RNA sequencing (RNA-seq) data, which quantified the gene expression values. With a standard deviation (SD) threshold of greater than 0.2, Perl software was used to extract the matrix of methylation sites located within − 2000 bp ~ + 500 bp of transcription start sites (TSSs), which covered 26,574 promoter region loci (11,247 for GAC and 15,327 for CAC) after filtering sites with missing values across the samples, on each chromosome (except for the sex chromosome). The methylation values were then adjusted with the R packages ‘impute’ and ‘sva’. Finally, Perl was used to merge the methylation site matrix with patient survival time and status data.

**Table 3:** Clinicopathologic features of patient with gastrointestinal adenocarcinoma.

Gastric adenocarcinoma (n = 406)		Colon adenocarcinoma (n = 385)	
Variables	N (%)	Variables	N (%)
**Age (years)**		**Age (years)**	
Mean ± SD	65.6 ± 10.9	Mean ± SD	67.0 ± 12.8
**Sex**		**Sex**	
Female	150 (36.9)	Female	180 (46.8)
Male	256 (63.1)	Male	205 (53.2)
**Grade**		**Grade**	
I	10 (2.5)	I	–
II	149 (36.7)	II	–
III	240 (59.1)	III	–
Unknown	7 (1.7)	Unknown	385 (100.0)
**Stage**		**Stage**	
I	56 (13.8)	I	66 (17.1)
II	118 (29.1)	II	151 (39.2)
III	167 (41.1)	III	103 (26.8)
IV	42 (10.3)	IV	54 (14.0)
Unknown	26 (6.4)	Unknown	11 (2.9)
**T stage**		**T stage**	
T1	23 (5.7)	T1	10 (2.6)
T2	85 (20.9)	T2	68 (17.7)
T3	185 (45.6)	T3	263 (68.3)
T4	103 (25.4)	T4	44 (11.4)
Unknown	10 (2.5)	Unknown	0 (0.0)
**N stage**		**N stage**	
N0	122 (30.0)	N0	231 (60.0)
N1	109 (26.9)	N1	88 (22.9)
N2	80 (19.7)	N2	66 (17.1)
N3	78 (19.2)	N3	0 (0.0)
Unknown	17 (4.2)	Unknown	0 (0.0)
**M stage**		**M stage**	
M0	361 (88.9)	M0	286 (74.3)
M1	27 (6.7)	M1	54 (14.0)
Unknown	18 (4.4)	Unknown	45 (11.7)

*SD* standard deviation.

### Consensus clustering analysis

With a criterion of *P* < 0.001, 177 prognosis-related promoter methylation sites (74 for GAC and 103 for CAC) and 131 independent prognostic methylation sites (68 for GAC and 63 for CAC) were identified via univariate and multivariate Cox regression analysis, respectively. The co-methylation patterns of these independent prognostic methylation sites were explored by correlation analysis, and visualized through the ‘corrplot’ package. Based on the independent prognostic methylation sites, the R package ‘ConsensusClusterPlus’, which provides quantitative and visual evidence of stability for measuring the number of unsupervised clusters in the dataset, was used for promoter methylation-based consensus clustering of GAC and CAC^44^. In addition, the K-means algorithm and cumulative distribution function (CDF) curve were applied to determine the best number of clusters. According to the CDF curve, the best and most stable number of clusters is the K value at which significant changes no longer occur. Moreover, 50 iterations (with 80% of the samples per iteration) with a variable of maxK = 9 were conducted for stable clusters. We plotted survival curves for each cluster according to promoter methylation-based clusters and compared site methylation levels in each subtype with heatmaps. To determine the methylation level between clusters, differential analysis of methylation sites in samples among the clusters was performed in R with a false discovery rate (FDR) threshold of < 0.05. The differential methylation sites were visualized with heatmaps (combining clinical parameters) and box plots with the packages ‘ComplexHeatmap’ and ‘reshape2′, respectively. Additionally, we accessed the MSI of each sample from TCGA database, and compared the MSI level between clusters. The R package ‘ggplot2′ was utilized to display the distribution of clinicopathologic features in clusters.

### Genes containing prognostic sites and molecular pathway enrichment analysis

Initially, genes containing independent prognostic methylation sites were identified by Perl, and their expression levels were visualized via unsupervised hierarchical clustering. To predict the potential functions of genes containing independent prognostic methylation sites, which were subjected to Gene Ontology (GO) biological process (BP) and Kyoto Encyclopedia of Genes and Genomes (KEGG) enrichment analysis with R packages (‘colorspace’, ‘stringi’, ‘ggplot2′, ‘clusterProfiler’, ‘org.Hs.eg.db’ and ‘enrichplot’).

### Identification of promoter methylation-based signatures

To search for hypo- or hyper-methylated sites to construct prognostic signatures, the hypo- or hyper-methylated sites from TCGA database were included in Lasso Cox regression analysis to generate prognostic scoring signatures,which could divide patients into high-risk and low-risk groups based on the mean risk score (RS) value. The RS was calculated as the sum of the products of locus methylation levels and coefficients, via the following formula:

$${\text{Risk}}\,{\text{Score}}\,\left( {{\text{RS}}} \right) = \sum\limits_{i}^{k} {\left( {Methi \times Coei} \right)}$$

where ‘i’ and ‘k’ represent the ‘i’th methylation locus and the number of methylation sites, respectively. To verify the efficiency, accuracy and independence of the signatures, Kaplan–Meier analysis and receiver operating characteristic (ROC) curves were used to evaluate the accuracy and prediction efficiency of signature. Univariate and multivariate analysis were used to explore the prognostic value of risk scoring signatures.

### Validation in separate cohorts

The DNA methylation profiles (Illumina Human Methylation 27 platform) of GAC and CAC were separately downloaded from the UCSC Xena platform (http://xena.ucsc.edu/). These two data sets were used as validation cohorts to verify the stability and mobility of methylation-based signatures.

### Nomogram construction

According to univariate analysis, the prognosis-related variables were enrolled into the training cohort with the ‘rms’ package as a medium, so as to participate in the construction of the nomogram for predicting 3-year and 5-year OS of patients. Afterwards, the ROC curve and calibration curve were used to evaluate the predictive performance and accuracy of the nomogram.

### Statistical analysis

Continuous variables were represented as mean ± standard deviation. χ^2^ test and T-test/variance analysis were separately used to compare the difference distribution of dichotomous variables and continuous variables. Survival analyse was conducted using Kaplan–Meier statistics and Log-rank tests. All statistical analyses were carried out in R and Perl softwares, and *P* < 0.05 was considered to be statistically significant.

## Supplementary information

Supplementary Information.

## Data Availability

The methylation profiles used in this study were derived from UCSC Xena platform (http://xena.ucsc.edu/). The transcription profiles were accessed from the Cancer Genome Atlas (TCGA, http://cancergenome.nih.gov/) database.

## Abbreviations

GAC: Gastric adenocarcinoma
CAC: Colon adenocarcinoma
OS: Overall survival
TCGA: The Cancer Genome Atlas
RS: Risk score
TNM: Tumor node metastasis
SD: Standard deviation
TSS: Transcription start site
CDF: Cumulative distribution function
GO: Gene ontology
BP: Biological process
KEGG: Kyoto Encyclopedia of Genes and Genomes
FDR: False discovery rate
