# Supplementary material for: Gastrointestinal adenocarcinoma analysis identifies promoter methylation-based cancer subtypes and signatures
Source: Sci Rep. 2020 Dec 4;10:21234. doi: 10.1038/s41598-020-78228-y (PMC7719188; doi:10.1038/s41598-020-78228-y)
Supplement: Supplementary file 1 — Supplementary Information. [file 41598_2020_78228_MOESM1_ESM.doc]

# Gastrointestinal adenocarcinoma analysis identifies promoter methylation-based cancer subtypes and signatures

Renshen Xiang^1^ & Tao Fu^*,1^

**Author affiliations:** ^1^Department of Gastrointestinal Surgery II, Renmin Hospital of Wuhan University, Wuhan, Hubei Province, China.

**Corresponding Author:**

*Tao Fu, MD. Department of Gastrointestinal Surgery II, Renmin Hospital of Wuhan University, Wuhan 430060, China. Email: tfu001@whu.edu.cn

**
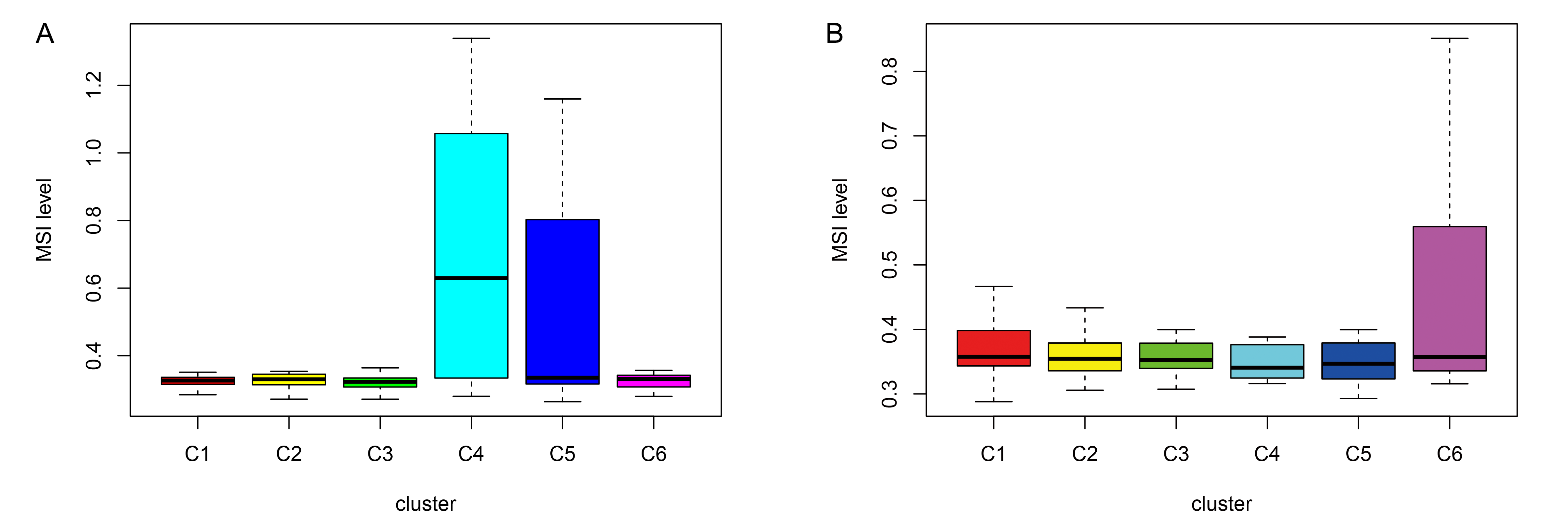
**

**Supplementary Figure 1. Microsatellite instability across clusters. (A)** In GAC, cluster 4 has the highest MSI, followed by cluster 5, while the other clusters showed a low MSI, including the hypo-methylated cluster 1. **(B)** cluster 4 showed the lowest MSI than the other five clusters, even if the difference in MSI between six clusters was not obvious. GAC: gastric adenocarcinoma, CAC: colon adenocarcinoma, MSI: microsatellite instability.

**Supplementary table 1.** Multivariate analysis of promoter methylation sites in GAC.

| **Sites** | **HR** | **HR.95L** | **HR.95H** | **P value** |
| --- | --- | --- | --- | --- |
| cg04586563 | 0.000222 | 7.91E-06 | 0.006218 | 7.53E-07 |
| cg05127924 | 0.020001 | 0.00405 | 0.098773 | 1.58E-06 |
| cg16085042 | 65019510 | 27741.22 | 1.52E+11 | 5.52E-06 |
| cg24619694 | 0.028655 | 0.006121 | 0.134153 | 6.47E-06 |
| cg16954341 | 0.085527 | 0.027978 | 0.26145 | 1.61E-05 |
| cg18849169 | 0.050443 | 0.01285 | 0.198017 | 1.86E-05 |
| cg26705561 | 0.096029 | 0.032732 | 0.281732 | 1.98E-05 |
| cg22572159 | 0.180841 | 0.081805 | 0.399773 | 2.39E-05 |
| cg04081402 | 0.002068 | 0.000117 | 0.036423 | 2.41E-05 |
| cg11251877 | 0.025873 | 0.00469 | 0.142749 | 2.74E-05 |
| cg22334665 | 0.104473 | 0.035584 | 0.306731 | 3.95E-05 |
| cg01139966 | 86.83229 | 10.30352 | 731.7739 | 4.05E-05 |
| cg15787039 | 0.076233 | 0.022244 | 0.261264 | 4.21E-05 |
| cg08942800 | 0.108157 | 0.036763 | 0.318199 | 5.35E-05 |
| cg11052143 | 0.146308 | 0.056591 | 0.378261 | 7.31E-05 |
| cg15701111 | 0.013834 | 0.001657 | 0.115503 | 7.70E-05 |
| cg14407437 | 0.116857 | 0.040293 | 0.338905 | 7.76E-05 |
| cg10730712 | 0.221935 | 0.104816 | 0.469919 | 8.39E-05 |
| cg14832904 | 0.034412 | 0.006356 | 0.186298 | 9.23E-05 |
| cg20727114 | 0.060977 | 0.014963 | 0.248494 | 9.53E-05 |
| cg12645220 | 0.123054 | 0.042761 | 0.354118 | 0.000102 |
| cg13449778 | 0.140511 | 0.051913 | 0.380311 | 0.000112 |
| cg25201363 | 0.05426 | 0.012324 | 0.238892 | 0.000117 |
| cg08359956 | 0.05143 | 0.01129 | 0.234271 | 0.000125 |
| cg18396533 | 0.078267 | 0.021257 | 0.288168 | 0.000128 |
| cg10971790 | 0.160724 | 0.062832 | 0.411133 | 0.000136 |
| cg10201668 | 0.012589 | 0.001323 | 0.119793 | 0.000141 |
| cg25047001 | 0.121022 | 0.040695 | 0.359905 | 0.000146 |
| cg14174099 | 0.201509 | 0.087963 | 0.461623 | 0.000152 |
| cg01722994 | 0.0794 | 0.02123 | 0.296954 | 0.000167 |
| cg20831708 | 0.126106 | 0.042833 | 0.371272 | 0.000171 |
| cg12619509 | 0.083131 | 0.022713 | 0.304268 | 0.000172 |
| cg02735486 | 0.066527 | 0.016138 | 0.274244 | 0.000177 |
| cg23442323 | 0.06669 | 0.016067 | 0.276809 | 0.000192 |
| cg08623787 | 0.174522 | 0.069415 | 0.43878 | 0.000206 |
| cg04915182 | 0.06458 | 0.015129 | 0.275663 | 0.000215 |
| cg15105703 | 0.060907 | 0.01383 | 0.268234 | 0.000216 |
| cg04595372 | 0.070315 | 0.017199 | 0.287474 | 0.00022 |
| cg00622677 | 0.145263 | 0.051713 | 0.408045 | 0.000251 |
| cg19614321 | 0.093001 | 0.025995 | 0.332729 | 0.00026 |
| cg00903242 | 0.216326 | 0.094868 | 0.493286 | 0.000272 |
| cg24625128 | 0.126959 | 0.04157 | 0.387748 | 0.000291 |
| cg18695917 | 0.152031 | 0.054713 | 0.422449 | 0.000303 |
| cg05203877 | 0.202624 | 0.08457 | 0.485471 | 0.000342 |
| cg09068528 | 0.156438 | 0.056411 | 0.433826 | 0.000364 |
| cg25356886 | 0.01182 | 0.001029 | 0.135829 | 0.000367 |
| cg11038843 | 0.104585 | 0.029495 | 0.370846 | 0.000472 |
| cg22775000 | 0.180505 | 0.069126 | 0.471342 | 0.000472 |
| cg18602314 | 0.126141 | 0.039508 | 0.402746 | 0.000473 |
| cg08632701 | 0.03854 | 0.006208 | 0.239253 | 0.000474 |
| cg09775312 | 0.193211 | 0.076781 | 0.486196 | 0.00048 |
| cg21625881 | 0.029514 | 0.004081 | 0.213458 | 0.000483 |
| cg14538332 | 0.133986 | 0.043297 | 0.414631 | 0.000488 |
| cg19564877 | 0.094129 | 0.024762 | 0.357815 | 0.000523 |
| cg11392765 | 0.085379 | 0.021254 | 0.342981 | 0.000524 |
| cg14899547 | 616238.8 | 324.0419 | 1.17E+09 | 0.000539 |
| cg06505666 | 887740.5 | 375.4579 | 2.1E+09 | 0.000549 |
| cg17398595 | 0.174035 | 0.064078 | 0.472679 | 0.000604 |
| cg25519930 | 0.194442 | 0.07626 | 0.495779 | 0.000605 |
| cg19568591 | 0.130422 | 0.040597 | 0.418991 | 0.000624 |
| cg02064106 | 0.240332 | 0.106135 | 0.544208 | 0.000628 |
| cg25437385 | 0.185762 | 0.070749 | 0.487741 | 0.000631 |
| cg16722536 | 0.088905 | 0.022089 | 0.357835 | 0.000658 |
| cg15425811 | 0.166235 | 0.059117 | 0.467446 | 0.00067 |
| cg07028533 | 0.211266 | 0.085717 | 0.520709 | 0.000731 |
| cg14135551 | 0.228389 | 0.096398 | 0.541106 | 0.000792 |
| cg11226328 | 12300.73 | 49.75346 | 3041153 | 0.000809 |
| cg24670715 | 0.126831 | 0.037401 | 0.43009 | 0.000919 |

GAC: gastric adenocarcinoma; HR: hazard ratio.

**Supplementary table 2.** Multivariate analysis of promoter methylation sites in CAC.

| **Sites** | **HR** | **HR.95L** | **HR.95H** | **P value** |
| --- | --- | --- | --- | --- |
| cg15786837 | 985.6655 | 76.21859 | 12746.71 | 1.30E-07 |
| cg07509155 | 7586.615 | 273.0882 | 210762.4 | 1.38E-07 |
| cg16711185 | 1.29E+11 | 7703790 | 2.15E+15 | 2.52E-07 |
| cg22190705 | 9516872 | 16777.13 | 5.4E+09 | 6.80E-07 |
| cg20888386 | 7599.097 | 203.1004 | 284323.7 | 1.33E-06 |
| cg15170424 | 323.5645 | 30.9725 | 3380.223 | 1.38E-06 |
| cg08617916 | 36305.34 | 485.8888 | 2712714 | 1.84E-06 |
| cg26411702 | 2712.827 | 103.6145 | 71027.04 | 2.08E-06 |
| cg23446109 | 6126.688 | 145.3504 | 258247 | 4.91E-06 |
| cg20070077 | 1.93E+22 | 5.24E+12 | 7.09E+31 | 4.97E-06 |
| cg17950095 | 6635.654 | 130.399 | 337670.6 | 1.14E-05 |
| cg18304195 | 25456508 | 11810.03 | 5.49E+10 | 1.34E-05 |
| cg06790324 | 4077.774 | 96.02297 | 173169.4 | 1.38E-05 |
| cg15796941 | 1635.361 | 57.63929 | 46399.02 | 1.46E-05 |
| cg21922841 | 34016279 | 13223.21 | 8.75E+10 | 1.50E-05 |
| cg09475324 | 1.79E+21 | 2.49E+11 | 1.29E+31 | 2.38E-05 |
| cg20822579 | 2195415 | 2505.206 | 1.92E+09 | 2.40E-05 |
| cg18530716 | 194.4206 | 16.45052 | 2297.761 | 2.88E-05 |
| cg01520924 | 2801.027 | 66.89347 | 117287.3 | 3.10E-05 |
| cg03977782 | 37087051 | 9628.991 | 1.43E+11 | 3.51E-05 |
| cg03600318 | 495.5048 | 22.1023 | 11108.57 | 9.19E-05 |
| cg03017653 | 242.2088 | 15.34013 | 3824.29 | 9.64E-05 |
| cg13812587 | 5908.192 | 74.39051 | 469236.4 | 1.00E-04 |
| cg23427565 | 1.84E+18 | 1.14E+09 | 2.98E+27 | 0.000101 |
| cg22764341 | 137.2351 | 11.26796 | 1671.419 | 0.000114 |
| cg22176895 | 538996.5 | 646.6607 | 4.49E+08 | 0.00012 |
| cg07138512 | 2280.198 | 43.75919 | 118816.3 | 0.000126 |
| cg05417950 | 87.20592 | 8.877945 | 856.6027 | 0.000127 |
| cg11326613 | 1469944 | 1031.68 | 2.09E+09 | 0.000127 |
| cg08317263 | 356.5717 | 17.61348 | 7218.526 | 0.000129 |
| cg15639045 | 7939.399 | 78.34408 | 804579.7 | 0.000139 |
| cg06471905 | 18656.91 | 118.3702 | 2940608 | 0.00014 |
| cg19779211 | 26233.21 | 136.6935 | 5034486 | 0.000149 |
| cg17329249 | 49.42194 | 6.502733 | 375.6156 | 0.000164 |
| cg13859324 | 44.14749 | 6.153382 | 316.7365 | 0.000165 |
| cg08704509 | 24729.86 | 124.9892 | 4892949 | 0.000177 |
| cg11833861 | 98047.29 | 237.0835 | 40548037 | 0.000185 |
| cg25070010 | 396008.6 | 424.7139 | 3.69E+08 | 0.00022 |
| cg24496666 | 590.6128 | 19.87259 | 17553 | 0.000227 |
| cg21212956 | 5694.154 | 56.87253 | 570106.3 | 0.000234 |
| cg13294594 | 150.5395 | 10.34787 | 2190.03 | 0.000242 |
| cg24206256 | 60.13873 | 6.734613 | 537.0267 | 0.000245 |
| cg09893305 | 20.52767 | 4.069865 | 103.5379 | 0.000252 |
| cg07293947 | 5989.662 | 50.23278 | 714196 | 0.000363 |
| cg27626299 | 13.00527 | 3.162568 | 53.48091 | 0.000377 |
| cg08461397 | 2.55E+15 | 8063418 | 8.09E+23 | 0.000382 |
| cg17808849 | 1.36E+16 | 16064525 | 1.15E+25 | 0.000397 |
| cg18397523 | 14.5526 | 3.295406 | 64.2647 | 0.00041 |
| cg07389922 | 39241.87 | 104.9505 | 14672865 | 0.000466 |
| cg09244244 | 134.3722 | 8.508306 | 2122.149 | 0.0005 |
| cg27020690 | 1002.781 | 20.15842 | 49883.32 | 0.000527 |
| cg10414946 | 51.01835 | 5.402593 | 481.782 | 0.000598 |
| cg26261431 | 1.95E+08 | 3524.313 | 1.08E+13 | 0.000614 |
| cg07777378 | 1.06E+22 | 2.31E+09 | 4.88E+34 | 0.000652 |
| cg05532892 | 5853.962 | 39.92782 | 858270.5 | 0.000652 |
| cg21610192 | 7.61E+20 | 6.03E+08 | 9.61E+32 | 0.000719 |
| cg14823109 | 3.98E+34 | 1.59E+14 | 9.91E+54 | 0.000885 |
| cg21468416 | 251.5503 | 9.660964 | 6549.817 | 0.000888 |
| cg06250108 | 2.1E+14 | 748513.9 | 5.90E+22 | 0.000891 |
| cg04569233 | 99.23479 | 6.559326 | 1501.304 | 0.00091 |
| cg14651992 | 3237.984 | 27.21943 | 385185.8 | 0.000916 |
| cg24127989 | 370.9064 | 11.11711 | 12374.75 | 0.000947 |
| cg26989531 | 1342823 | 306.4143 | 5.88E+09 | 0.000973 |

CAC: colon adenocarcinoma; HR: hazard ratio.

Supplementary table 3. Difference analysis of independent prognostic promoter methylation sites in GAC.

| **Sites** | **Log_10_(FC)** | **P Value** | **FDR** |
| --- | --- | --- | --- |
| cg14174099 | -3.434239831 | 1.07E-19 | 7.26E-19 |
| cg22775000 | -2.713527385 | 1.88E-16 | 6.10E-16 |
| cg05203877 | -2.698459863 | 1.09E-20 | 1.06E-19 |
| cg14135551 | -2.607930482 | 2.28E-19 | 1.41E-18 |
| cg11392765 | -2.507804931 | 2.69E-10 | 3.89E-10 |
| cg24625128 | -2.325490166 | 4.81E-16 | 1.42E-15 |
| cg13449778 | -2.303539683 | 2.32E-17 | 9.29E-17 |
| cg18602314 | -2.287139678 | 4.54E-19 | 2.57E-18 |
| cg00622677 | -2.271159538 | 2.09E-08 | 2.64E-08 |
| cg18695917 | -2.233513697 | 2.12E-15 | 4.64E-15 |
| cg09068528 | -2.093544137 | 1.54E-11 | 2.82E-11 |
| cg10971790 | -2.002503431 | 2.55E-11 | 4.34E-11 |
| cg25047001 | -1.966163019 | 1.09E-16 | 3.71E-16 |
| cg22572159 | -1.966061094 | 3.59E-22 | 1.22E-20 |
| cg23442323 | -1.878605638 | 2.37E-14 | 4.60E-14 |
| cg00903242 | -1.871429583 | 6.56E-22 | 1.49E-20 |
| cg14538332 | -1.864952623 | 3.08E-20 | 2.62E-19 |
| cg25519930 | -1.809828286 | 1.71E-18 | 8.27E-18 |
| cg12645220 | -1.747639792 | 3.88E-20 | 2.93E-19 |
| cg01722994 | -1.739293477 | 7.31E-15 | 1.46E-14 |
| cg10730712 | -1.699984384 | 7.91E-21 | 8.97E-20 |
| cg19564877 | -1.630459044 | 1.95E-06 | 2.33E-06 |
| cg09775312 | -1.617784044 | 1.38E-10 | 2.09E-10 |
| cg08623787 | -1.495822299 | 2.06E-21 | 2.80E-20 |
| cg02064106 | -1.472325319 | 1.91E-15 | 4.49E-15 |
| cg07028533 | -1.461897409 | 1.82E-18 | 8.27E-18 |
| cg25437385 | -1.382205373 | 1.34E-15 | 3.64E-15 |
| cg11038843 | -1.355878887 | 9.41E-22 | 1.60E-20 |
| cg22334665 | -1.299566919 | 2.79E-17 | 9.97E-17 |
| cg04915182 | -1.297786341 | 1.05E-09 | 1.48E-09 |
| cg15105703 | -1.242391415 | 2.57E-10 | 3.80E-10 |
| cg17398595 | -1.112788232 | 2.46E-16 | 7.59E-16 |
| cg19614321 | -1.106906352 | 2.93E-15 | 6.23E-15 |
| cg19568591 | -1.053934484 | 6.24E-11 | 9.86E-11 |
| cg15425811 | -0.990807518 | 5.64E-16 | 1.60E-15 |
| cg16722536 | -0.96780459 | 3.25E-18 | 1.38E-17 |
| cg16954341 | -0.940871192 | 2.58E-17 | 9.76E-17 |
| cg11052143 | -0.907656955 | 3.18E-22 | 1.22E-20 |
| cg14407437 | -0.679024915 | 1.46E-07 | 1.77E-07 |
| cg08359956 | -0.652438654 | 1.22E-05 | 1.43E-05 |
| cg18396533 | -0.571813857 | 6.09E-15 | 1.26E-14 |
| cg20727114 | -0.57025761 | 0.010500934 | 0.011334342 |
| cg20831708 | -0.526213098 | 1.81E-15 | 4.39E-15 |
| cg02735486 | -0.518570525 | 3.24E-11 | 5.38E-11 |
| cg08942800 | -0.503875131 | 1.09E-10 | 1.69E-10 |
| cg04595372 | -0.500543837 | 1.44E-15 | 3.76E-15 |
| cg25201363 | -0.497543239 | 8.30E-19 | 4.34E-18 |
| cg10201668 | -0.489736637 | 2.13E-11 | 3.71E-11 |
| cg26705561 | -0.446852869 | 1.81E-15 | 4.39E-15 |
| cg24670715 | -0.422834749 | 1.83E-09 | 2.49E-09 |
| cg15787039 | -0.363332431 | 1.48E-05 | 1.71E-05 |
| cg12619509 | -0.359817047 | 1.44E-07 | 1.77E-07 |
| cg18849169 | -0.349947302 | 2.00E-15 | 4.53E-15 |
| cg21625881 | -0.276865377 | 2.13E-11 | 3.71E-11 |
| cg24619694 | -0.256504186 | 5.88E-11 | 9.52E-11 |
| cg05127924 | -0.238586761 | 5.54E-09 | 7.24E-09 |
| cg25356886 | -0.223763231 | 1.21E-12 | 2.28E-12 |
| cg15701111 | -0.179785202 | 3.99E-09 | 5.31E-09 |
| cg14832904 | -0.13865854 | 0.00095965 | 0.001087604 |
| cg08632701 | -0.122879442 | 8.44E-09 | 1.08E-08 |
| cg11251877 | -0.117551955 | 0.001047747 | 0.00116798 |
| cg04081402 | -0.111806023 | 1.56E-09 | 2.16E-09 |
| cg04586563 | -0.034616163 | 0.02158402 | 0.022933022 |
| cg11226328 | 0.249059398 | 0.043154229 | 0.045145963 |
| cg01139966 | 1.213431572 | 0.007385866 | 0.008100627 |

GAC: gastric adenocarcinoma.

**Supplementary table 4.** Difference analysis of independent prognostic promoter methylation sites in CAC.

| **Sites** | **Log_10_(FC)** | **P Value** | **FDR** |
| --- | --- | --- | --- |
| cg20822579 | 1.003647143 | 0.012271 | 0.040688053 |
| cg07509155 | 1.042932088 | 0.000407421 | 0.004277916 |
| cg18304195 | 1.056673567 | 0.001059227 | 0.00834141 |
| cg03977782 | 1.153981495 | 0.000505258 | 0.00454732 |
| cg05417950 | 1.187953996 | 0.014662349 | 0.043987047 |
| cg22764341 | 1.474587662 | 0.00038823 | 0.004277916 |
| cg24127989 | 1.612412688 | 0.001722678 | 0.009866244 |
| cg21212956 | 1.693790469 | 0.006031065 | 0.025330474 |
| cg01520924 | 1.772098279 | 0.003822369 | 0.020067437 |
| cg08317263 | 1.785154172 | 0.001238961 | 0.00867273 |
| cg15639045 | 1.899671147 | 0.001446505 | 0.009112979 |
| cg15170424 | 2.090418238 | 0.000407421 | 0.004277916 |
| cg18530716 | 2.09820697 | 0.007448818 | 0.02932972 |
| cg15786837 | 2.693969319 | 2.69E-06 | 0.00016921 |
| cg24206256 | 2.928975256 | 5.95E-06 | 0.000187546 |
| cg17329249 | 3.35549283 | 5.85E-05 | 0.001227892 |
| cg09893305 | -1.253510841 | 8.95E-15 | 5.64E-13 |
| cg18397523 | 2.6746535 | 8.99E-09 | 3.52E-07 |
| cg27626299 | 2.214155681 | 1.12E-08 | 3.52E-07 |

CAC: colon adenocarcinoma.
